# Goals of care or goals of life? A qualitative study of clinicians’ and patients’ experiences of hospital discharge using Patient-Oriented Discharge Summaries (PODS)

**DOI:** 10.1186/s12913-020-05541-7

**Published:** 2020-07-24

**Authors:** Nayantara Hattangadi, Paul Kurdyak, Rachel Solomon, Sophie Soklaridis

**Affiliations:** 1 Center for Addiction and Mental Health, 33 Russell Street, 2nd floor, room 2059, Toronto, ON Canada; 2 The Hospital for Sick Children, Toronto, ON M5S 1S2 Canada

**Keywords:** Quality improvement, Mental health, Discharge planning, Patient experience, Provider experience, Qualitative research

## Abstract

**Background:**

Recognizing the need for improved communication with patients at the point of hospital discharge, a group of clinicians, patients, and designers in Toronto, Canada collaborated to develop a standardized tool known as the Patient-Oriented Discharge Summary (PODS). Although quantitative results suggest PODS helps mitigate gaps in knowledge, a qualitative inquiry from the clinician and patient perspective of psychiatric hospital discharge using PODS has not been widely explored. Our aim was to explore clinicians’ and patients’ experiences with PODS.

**Methods:**

We used a qualitative thematic analysis to explore clinicians’ (*n* = 10) and patients’ (*n* = 6) experiences with PODS. We used convenience sampling to identify and invite potential participants at the Center for Addiction and Mental Health in Toronto, Canada to participate in semi-structured interviews between February 2019 and September 2019. Data were analyzed using a thematic analysis approach to develop descriptive themes.

**Results:**

Emerging themes from the data between clinicians and patients were both different and complementary. Clinicians described PODS using the concept of “goals of care.” They relayed their experiences with PODS as a discrete event and emphasized its role in meeting their “goals of care” for discharge planning. Patients provided more of a “goals of life” perspective on recovery. They characterized PODS as only one facet of their recovery journey and not necessarily as a discrete or memorable event. Patients focused on their outcomes post-discharge and situated their experiences with PODS through its relation to their overall recovery.

**Conclusions:**

PODS was experienced differently by clinicians and patients. Clinicians experienced PODS as helpful in orienting them to the fulfillment of goals of care. Patients did not experience PODS as a particularly memorable intervention. Due to the information advantage that clinicians have about PODS, it is not surprising that clinicians and patients experienced the PODS differently. This study expanded our understanding of hospital discharge from clinicians and patients perspectives, and suggests that there are additional areas that need improvement.

## Background

The period following hospital discharge can be one of uncertainty for patients. Critical information about medications, monitoring one’s health, and when to seek emergency care is often omitted during discharge [1–3]. The information that is communicated often uses language that is beyond the literacy level of the patient or their primary caregiver, further exacerbating their comprehension, fatigue and memory during hospital discharge [4–6]. Consequently, patients often leave the hospital with an incomplete understanding of their diagnosis, treatment plan, and expected or concerning symptoms [7]. For patients discharged from inpatient psychiatric hospitalizations, adequate transitional support is especially important as many experience additional challenges to their mental health and wellbeing [8].

The need for effective communication during discharge is all the more pressing given that patients who have recently been discharged from the hospital are at a significant risk of experiencing adverse events, otherwise known as complications or injuries resulting from their treatment [9]. Reactions to medication and therapeutic errors are cited as some of the most common causes of adverse events [10]. Adverse events can lead to additional hospital visits, new and/or worsening symptoms, temporary or permanent disabilities, and death [11, 12]. Estimates of the number of patients who experience adverse events following discharge in Canada range from 7.5 to 23%, with over a quarter of these cases being deemed preventable [9, 13]. Effective communication of health-related information during hospital discharge is thus essential to mitigating the effects of and, where possible, preventing adverse events, as well as ensuring overall continuity of care after hospital discharge.

Recognizing the need for improved communication with patients at the point of discharge, a collaboration of clinicians, patients, and designers developed a standardized tool known as the Patient-Oriented Discharge Summary (PODS) [6]. PODS was created with the intention of working in a range of clinical environments (i.e. acute care, rehabilitation, surgery, etc.) and based on collaborators’ recommendations, developers structured PODS to communicate five pieces of health-related information: (1) when to take medications, (2) possible symptoms and what to do if they arise, (3) changes to routine and their duration, (4) future appointments and contact information, and (5) additional resources for information [10]. Developers sought to maximize patients’ comprehension of this information by keeping language at a fifth- or sixth-grade level, including images, and providing patients with space to take notes. An initial evaluation found that PODS fit well within existing discharge practices and resulted in improved patient and provider experiences, with 75% of clinicians and 95% of patients expressing that PODS would provide helpful information during discharge [6].

In 2015, a pilot study was conducted to test PODS across eight non-psychiatric hospitals in Toronto [14]. Patients who received PODS during their discharge from the hospital cited improvements in their knowledge, as over 95% reported that they knew the purpose and use of their medications, of side-effects and what to do if they arose, and of their scheduled follow-up appointments. Further, over 88% reported knowing when to return to their regular routines and who to contact for more information [14]. Of the clinicians who used PODS, approximately 90% thought the form was easy to use and would help patients and 80% reported that it did not add to their workload [14]. The pilot project results suggested that PODS can help mitigate gaps in comprehension for patients being discharged from medical hospitalization, with minimal added work for healthcare clinicians.

The issues related to discharge from psychiatric hospitalizations are similar – the need for information from clinicians to patients prior to discharge to facilitate the successful transition from a hospital setting to the community. Prior research on transition planning interventions among psychiatric patient populations suggests that discharge planning is key to preventing readmission [8]. Existing literature cites collaborative care between inpatient staff, psychoeducation for patients and caregivers, communication between inpatient and outpatient networks, and medication reconciliation as important pieces of effective discharge and transition planning interventions [15–17]. Therefore, the PODS tool for medical discharge was modified at the Centre for Addiction and Mental Health (CAMH), a tertiary care psychiatric hospital in Ontario, Canada, to suit the informational needs of individuals transitioning from psychiatric hospital to the community. Modifications included changing the language around patients’ purpose of admission to less diagnosis-centered i.e. from “I am here because I have XXX” to “I am here because I felt XXX”; providing space to add information about patient goals, community help to achieve goals, and supports after discharge.

The objective of this study was to evaluate patients’ and clinicians’ experience with this newly developed tool for psychiatric discharge. Given that PODS was a hospital initiative to be used by clinicians for patient discharge, we recognize that clinicians have more knowledge than patients about the application of the tool in practice [18]. Despite these drawbacks, we believe that it does not detract from the patient’s ability to describe the potential benefits of PODS during the discharge planning process and recognize that patients provide a unique view on the shortcomings of services that clinicians do not necessarily see. Patients can still be beneficiaries of the standardization of the discharge process that occurs as a result of the completion of PODS. Since PODS is still considered a work in progress, information from patients and clinicians can help to widen understandings about how these groups experience discharge using PODS, and inform further improvements to the tool.

## Methods

We used a qualitative thematic analysis and constructivist approach to answer our research question: What are clinicians’ and patients’ experiences with PODS? [19] Our aim was to gain insight into contextual factors that might affect its use and to elicit feedback on how PODS can be improved for future clinicians and patients. Social constructionism provides a theoretical basis for understanding how realities and views of the world are created by individuals through interactions with one another [20].

Instead of using an established theory or allowing the research to be guided by hypotheses, the study was designed to answer the research question by allowing the interactions between interviewer and participants to shape the data collection and analysis (inductive approach). Although we had an interview guide, the interviewer, depending on the flow of each interview, might have asked additional questions not included on the guide, to provide space for the interviewee to expand on their answers, or follow up on something that the interviewee might have said that was unexpected or interesting to the topic of discharge planning. We used the COREQ checklist (provided in supplementary information) to guide our reporting of this qualitative research study [21]. This study was approved by the CAMH Research Ethics Board (101–2018). We obtained verbal and written consent from all participants in this study.

### Setting and recruitment

We used convenience sampling to identify potential participants (clinicians and patients) between February 2019 and September 2019 at CAMH in Toronto, Canada [22]. CAMH is Canada’s largest mental health teaching hospital and one of the world’s leading research centers. CAMH’s clinical and research focuses include, but is not limited to, assessment and treatment of mood and anxiety disorders, schizophrenia, and addictions (alcohol, drugs and problem gambling).

Our eligibility criteria included clinicians who had discharged a patient using PODS, and English-speaking patients who had been discharged from inpatient units and received PODS. All clinicians who utilize PODS (physicians, social workers and pharmacists) were eligible to participate. Clinicians and patients from all inpatient units except the Emergency Assessment Unit (a short-stay, holding bed unit where PODS was not implemented) and Forensic (clients with serious mental illnesses who have come into contact with the law, and where PODS was not implemented) were eligible to participate. We did not specify or ask potential participants to disclose health or diagnosis information beyond being involved in the PODS process because their diagnosis was not going to be integrated into the results of this exploratory study.

We recruited clinicians by seeking assistance from the leadership teams of various inpatient units. Unit managers informed their staff about our study, and interested clinicians reached out to us to participate in the study. To recruit patients, we asked clinicians to provide an information letter during the discharge planning process to patients so that patients, if interested, could contact the research team. Study flyers were also distributed to the Outpatient Services to aid with recruitment of patients who had recently discharged from CAMH inpatient units. Patients were informed that their decision to participate would not impact present or future care received.

### Sample size

Our sample size was determined a priori using the five considerations outlined under the concept of information power [23]. These considerations ask the researcher to reflect on the study aim, sample specificity, theoretical background, quality of dialogue and strategy for analysis. Recognizing that our study was exploratory, our aim was narrow with a specific study population (clinicians and patients from a psychiatric hospital); the interviews were semi-structured and conducted by the lead author; and the analysis strategy included an in-depth analysis of participants’ narratives. Steered by these considerations, we determined that a sample of 16 participants provide sufficient information power for an exploratory study, and to capture the experiences of clinicians’ and patients’ experiences of hospital discharge using PODS.

### Data collection

All interested and eligible participants (clinicians and patients) were invited to participate in an in-person interview at CAMH or a telephone interview. Interviews with patients were completed between 8 and 12 months after discharge. Patients were recruited with assistance from clinicians and Outpatient Services; therefore, many patients who expressed interested to participate in interviews had varying discharge dates. Individual interviews (~ 45–50 min) using semi-structured interview guides were conducted to gain a fulsome understanding of clinicians’ and patients’ experiences of hospital discharge using PODS. Interview guides (provided in supplementary information) were based on a thorough review of the literature and keeping in line with the research question and objectives of the study.

Individual interviews were conducted to provide both participant groups with the opportunity to share their personal experiences, including positive or negative opinions of PODS, adherence to discharge and PODS instructions, and possible barriers to using PODS, in a confidential environment. An honorarium of $30 (cash) was offered to patients for their participation.

### Analysis

We used the thematic analysis approach of Braun and Clarke to review the transcribed interviews, generate codes, and develop descriptive themes [24]. Data analysis started by having the audio recordings transcribed verbatim by a professional transcriber and relocated into NVivo 11. Then, the transcripts were coded line-by-line and underwent several in-depth reviews by the lead author (NH) and the principal investigator (SS), resulting in the development of numerous open codes. Subsequently, the open codes were collapsed and grouped into forming descriptive categories. Finally, categories were refined and converged to create central themes. The themes were provided to two members of the research team (PK and RS) for further feedback and refinement. The research team engaged in consistent and iterative dialogue throughout the entire coding process to ensure that themes were not generated from a few vivid examples but instead that the process and resulting themes were thorough, inclusive, comprehensive and reflective of the entire data set.

## Results

A total of 10 clinicians and 6 patients from a wide range of inpatient units were included in the study. Sample characteristics are presented in Table 1.

**Table 1:** Sample Characteristics

Participant	ID	Gender	Unit	Position
Clinician	SP 01	F	General Psychiatric & Acute Care	Social Worker
	SP 02	F	General Psychiatric & Acute Care	Social Worker
	SP 03	F	General Psychiatric & Acute Care	Social Worker
	SP 04	M	General Psychiatric & Acute Care	Social Worker
	SP 05	M	Early Psychosis/Youth	Social Worker
	SP 06	F	Woman’s Inpatient	Social Worker
	SP 07	F	Early Psychosis/Youth	Social Worker
	SP 08	F	General Psychiatric & Acute Care	Social Worker
	SP 09	F	Woman’s Inpatient & General Psychiatric	Pharmacist
	SP 10	F	Woman’s Inpatient, General Psychiatric & Psychiatric Intensive Care	Pharmacist
Patient	SU 01	M	Early Psychosis	
	SU 02	M	Early Psychosis	
	SU 03	F	Early Psychosis	
	SU 04	F	Woman’s Inpatient	
	SU 05	M	General Psychiatric	
	SU 06	M	General Psychiatric	

**Fig. 1:**
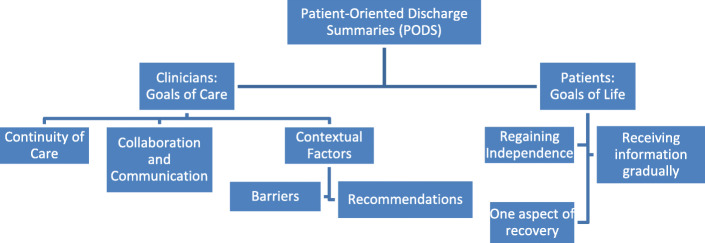
Results

Two overarching themes emerged which were different and complementary among the experiences of clinicians and patients with PODS (Fig. 1). Clinicians relayed their experiences with PODS as a discrete event and emphasized its role in meeting their “goals of care” for discharge planning (including the concepts of continuity of care and collaboration and communication). Given the role of PODS in the clinical environment, clinicians were able to describe the contextual factors that created barriers to its use and offer recommendations for improving its application.

Patients described PODS as only one facet of their recovery journey; they focused on their outcomes post-discharge and situated their experiences with PODS through its relation to their “goals of life” (including the concepts of regaining independence, receiving information gradually, and viewing discharge as one aspect of recovery).

### Clinicians: goals of care

#### Continuity of care

Clinicians discussed PODS as a tool that facilitates and organizes the discussion around care after discharge from the inpatient environment. The use of PODS provided an interface to discuss the goals of care and the continuity of care. Clinicians described PODS as a ‘one stop shop’ – a tool that highlights the most important information to impart to patients leaving their care. Namely, they discussed PODS as a tool that helps clinicians focus on having all the information in one place; specifically, what the patient needs and what they could benefit from post-discharge. Bearing in mind the overwhelming nature of inpatient discharges, clinicians felt that a consolidated take-away package is the most effective way to deliver critical information to discharging patients.

Considering that many patients go from inpatient units to outpatient services, clinicians discussed PODS’ utility in facilitating continuity of care, acknowledging that, if prepared well, PODS includes details for upcoming appointments and referrals for support services, “It [PODS] consolidates all the information – it can be overwhelming to get different pieces of paper, so it’s nice to have one package” [SP 09]. Clinicians perceived that PODS is beneficial for family members and caregivers, because individuals leaving CAMH are often managing illnesses that can impact their ability to comprehend their diagnosis and treatment plan:

It’s also helpful when the clients are confused and not completely well – so, at least the family has something and is getting a point of contact and has something to take away... Families really appreciate these documents – a lot of times the clients don’t really want to hold onto this paper, so family members are happy to get it to be looped in. [SP 05]

Given that patients can be vulnerable during this time, clinicians stated the importance of having caregivers in the community that are up-to-speed on critical pieces such as when to seek further care. For effective continuity of care post-discharge, clinicians felt that the information given to patients on their way out must be a concise but informative snapshot of their stay in the hospital and next steps.

#### Collaboration and communication

Effective collaboration and clear communication among team members have always been fundamental elements of interprofessional work. PODS was described as strengthening interprofessional collaboration among colleagues. Clinicians indicated that the introduction of PODS significantly increased communication on the unit, and facilitated collaboration between health care clinicians involved in the discharge process. Clinicians asserted that for PODs to be done well, communication within the team must be prioritized, “Everybody understands the importance of PODS – we’re all on the same page and we all know that this needs to get done” [SP 07]. With designated roles for physicians, pharmacists and social workers, from start to finish, PODS is a product of many clinicians working together. Clinicians felt that the implementation of PODS has impacted how clinicians are communicating to ensure procedures run as smoothly as possible. Moreover, the ease of document accessibility was mentioned as an added benefit, particularly when resources are limited, “It’s forced us to improve communication…the idea that everyone can access it is really helpful especially when you have a gap in clinician coverage” [SP 09].

Clinicians also discussed the consequences of miscommunication and disruptions to the systematic workflow; often, workflow miscommunications result in patients leaving the hospital without receiving the PODS that has already been created for them. This was particularly a concern on units with staff shortages, as temporary, relief or part-time workers are not adequately trained in PODS, and are thus unfamiliar with the process:

[Clinicians who are not here] full-time might not know the process, so they think that after seeing the doctor, the patient can leave; they don’t know its doctor➔pharmacist➔social worker (who gives PODS). [SP 02]

With various people involved in the process at different levels, clinicians agreed that the coordination of all the pieces of PODS can be challenging. Clinicians were candid that a lack of communication can and often does result in inefficient handoffs, chaos and frustration with the PODS process.

#### PODS in the clinical environment: contextual factors

Although clinicians acknowledged that PODS was developed to maintain uniformity and add structure to discharge-related processes and communication, they felt that an essential goal of their care was to contextualize each patient discharge. They described the importance of finding a meaningful balance between standardizing and individualizing instruction. Subscribing to the notion that ‘one size does not fit all’, clinicians discussed contextual factors that impact PODS’ utility and the extent to which it serves as a beneficial tool.

Specifically, some clinicians pointed to certain sections of the form that can feel unsuitable given patient specifics, and voiced the desire for flexibility to take out sections of the form that are not relevant. While a standardized tool serves as a useful guide that keeps everyone on the same page, the needs of each patient, and the situation surrounding their discharge, vary:

I have a patient who’s leaving today. He’s like carrying a garbage bag, his shoes are

everywhere, like I know he’s going to lose this or throw it out in five seconds of him

being out the door/potentially never read it. I could barely get him to like look over it with me. I’ve got a window of like four seconds to talk to this guy before he’s going to erupt and bust out the door as he’s done earlier in the admission. I’m like this is the medication you need, like remember that. Sometimes you really just need in this type of setting. [SP 01]

Clinicians discussed that under many instances, it can feel condescending and inappropriate to utilize a tool with pre-planned headings particularly if they have not been admitted long enough to include any information under a specific section or if they’re discharging a patient who had a hostile admission and is not interested in receiving PODS. They also commented on the length of the tool and felt that sometimes it is most effective to focus on the most useful sections, instead of attempting to tackle all of them:

The way that it’s standardized it doesn’t work for everyone…There are some patients who are going to crumple it up right away and aren’t going to need it. There are some people who are completely fed up and you don’t really need to put much on the form. [SP 02]

Because situations and contextual factors can vary greatly, clinicians emphasized that they are in the best position to make this judgment call, as they have the strongest understanding of their patients and their needs:

There’s something to be said for clinical opinions and knowing the client and what’s going to work best for them...otherwise it can feel like we’re forcing people to take all of this exact information…which is maybe not therapeutic in every case” [SP 03].

The implementation of PODS is a template that does not apply to all circumstances. Clinicians used their clinical reasoning to adapt PODS to their patients’ individual needs.

#### Barriers

Clinicians identified a series of barriers that impede their use of PODS; most notably, they pointed to a lack of resources and time constraints. While social workers were identified as the chief drivers of discharge (as they typically tie all the pieces together and are the final deliverer of PODS to the patient), clinicians identified a lack of social work resources on units as a systemic issue:

We’re under-staffed in terms of social work. So, PODS is a social work task and we’ve not been able to do it as often and then people are tracking the PODS. So, I’ve had people come up to me and be like, so PODS isn’t being done for every patient. No, it’s not, because we don’t have the social work resources to do it right now. Not in terms of we don’t want to do it, just in terms of we can’t get to every single thing. Especially if it’s such a high turnover and fast paced…And then, the frustration is usually just 16 things going on and you have to wait and be the last person to print this out. [SP 03]

Resource and time constraints were echoed by pharmacists as well, who are often covering multiple units due to staff shortages, “If the pharmacist is away, there’s a desk pharmacist who covers just the orders, but they wouldn’t be necessarily assisting with the PODS just because we don’t have the person power for that” [SP 09]. In addition, clinicians shared frustrations with navigating their growing administrative responsibilities, particularly with inputting the same information in multiple places and working with systems that could be run more efficiently if programs were designed to ‘speak better to each other’:

Clinicians acknowledged that time pressures and increasing workloads affect whether the process occurs in the way it was designed to: “be done in conjunction with the patients. I think the idea is you’re supposed to go sit down and fill things out together. But, I don’t have time to do that… This is such a lovely, ideal world kind of a thing. It does not happen” [SP 03].

Although clinicians expressed that they want to spend more time getting to know their patients well enough to assist in crafting an informative discharge summary with information that’s helpful to them, the reality was that discharges are put together last minute and this means there are many missed opportunities to use PODS to its full potential. Most clinicians asserted that they remain hopeful that they are doing their best, while acknowledging that with the limited resources at their disposal, it is not going to be a perfect discharge for every patient.

#### Recommendations

Clinicians proposed three practical recommendations to improve PODS. First, they suggested appointing PODS specific champions or ‘super-users’ within each unit to ensure staff are engaged, trained and motivated to complete PODS, “Finding champions, I think it’s all like project management and things like that, finding the people who are engaged” [SP 09].

Second, clinicians recommended that a flagging system could be implemented to streamline workflow. Since PODS relies on methodical collaboration between various health care clinicians, an automated flagging system that prompts the next clinician in the process to complete their portion of the form could streamline the workflow:

Finding out when the med-rec [medication reconciliation] is done is difficult. There’s no automated way, so it’s usually an email. It would usually be the physician with the resident, if they have one, myself, and the pertinent social worker on an email, being like, please let me know when the med-rec is done. The med-rec is done, so my part is done ready for PODS…Having some sort of flagging system would be helpful. [SP 09]

Third, clinicians drew attention to the many technological glitches within the PODS system. Understandably, navigating rigid structures and troubleshooting technical errors in the system on a daily basis causes frustrations and affects the time and resources spent on populating PODS:

If the pharmacist hasn’t done her part, then the medications show up all wonky and weird. So, I think I have to wait for her to be done in order to put in all the rest of my stuff. I just think that if the process could change that no matter when the medications are done or no matter when I’ve kind of fixed all of my part, then the document could be created from the pharmacist’s end or from whoever’s end really [SP 01].

The formatting malfunctions were pinpointed as a central cause for time wastage. With limited to no availability to manually restructure the document and fix the formatting issues, clinicians discussed often having no choice but to deliver a less-than user-friendly document to their patient.

### Patients: goals of life

Patients discussed their experiences with PODS by referencing their goals of life, including regaining independence out of the hospital, receiving information gradually, and viewing discharge as one aspect of their recovery. Discussions also led to identifying overarching barriers in the way of exploring patient experiences with PODS.

#### Regaining Independence

Acknowledging that leaving the hospital can be very difficult, patients lay emphasis on the importance of tools that can assist with the transition out of the hospital, while also helping to organize patients’ personal goals. Patients highlighted (re)gaining independence as a goal of life, and felt PODS does well in assisting them with assembling important information – specifically, their next steps (including outpatient appointments, medication management plans and others), “There was a set of instructions for my follow-up appointments, and that’s useful, to just have all the follow-up appointments listed out at least for the next month. It keeps things nice and organized” [SU 06]. Patients appreciated when PODS was designed in a way that outlines steps to meet specific goals, “I want to be in a place where I can work again, trying to make it on my own…They gave me a lot of resources, connected me to like the next step…I wanted to go back to work and I wanted to like have some level of independence and that was my main goal” [SU 05]. Patients expressed that PODS kept them on track during a particularly confusing time, “It was very clear…it reminded me that there were things to do after I left” [SU 04]. PODS served as an important reminder for many of the discussions that may have happened during their stay in the hospital and summarized what steps needed to be taken to manage their health post-discharge.

#### Receiving information gradually

For all patients, recalling their discharge period during the interviews was challenging because their memory of it was relatively vague. Hence, patients asserted that PODS helped provide necessary structure, clarity and management during a time of chaos and confusion. Agreeing that overloading patients with instructions at the very end of their stay is overwhelming, they emphasized that the gradual imparting of information is crucial and most helpful to prepare patients for discharge.

Patients also shared feeling apprehensive and restless towards the end of their stay, as well as that the days and weeks after discharge. To alleviate this stress, they suggested developing a system of check-in calls from members of the care team, to help transition patients out of the hospital.

Here, you're pampered, and you're taken care of, and all those things, and when you're discharged, you're basically on your own in your head…First few days can be very confusing when you leave this place, and then the next morning you wake up on your own and make your breakfast. All these things can be very confusing….Maybe having a worker that will just make a phone call and say, hi, I just want to make sure that you got there safely and you're doing okay, and, I don't know, reminds you that your appointment with your doctor is on Thursday, that’s it. Maybe something like that. [SU 03]

In addition, their suggestion to implement post-discharge check-in calls as a means of keeping communication channels open was described as providing reassurance for patients who are feeling overwhelmed with all they have to do, “Even over the phone or something where I connect with someone and I give them this is what I’m doing, this is what I need to do in order to stay on track. And then can kind of every six months or something give me … just do a check in and see if I’m still there or if there’s any issues” [SU 05]. Patients also expressed that introducing check-in calls serves as an opportunity for care clinicians to reassess whether goals and needs have changed, and remind patients about helpful resources, “Like someone there to check in, someone there to listen, maybe provide you with more resources if you need it” [SU 02].

Moreover, implementing a practice of connecting patients post-discharge to individuals who act as navigators for resources, such as employment and networking systems, can help people succeed with their goals of life once they’ve left the hospital. This would be particularly valuable for patients without additional supports available in their communities, “Having a social worker or occupational therapist or someone who can give you some advice, give you some input for people who don’t have families or don’t have anyone else to look to for support. That point of connection is going to be more important so having something like that would be awesome” [SU 03]. Ultimately, patients emphasized that it was meaningful for them to feel that members of their care team were thinking of them, and were invested in making sure everything was going as anticipated.

#### Discharge as one aspect of recovery

Patients viewed discharge as one aspect of their recovery and faced difficulties in discussing barriers and recommendations for PODS due to a lack of understanding of the overarching system. They identified that they had no reference points to critique the process or tool, and no way of commenting on what could have been more helpful because they only knew what they received. Patients’ capacity to describe experiences during hospital discharge was further challenged due to recall issues, “I had a little bit of difficulty following people’s speech and remembering the words that they were saying to me…had a hard time listening to people, and understanding people…like cognitive disorganization” [SU 01].

In addition, their illness made it difficult to participate in the process, “It was very confusing to know what actually happened and what actually didn’t happen, and what is the disease, what does it mean to have a bipolar disorder, what does it mean?” [SU 03]. Many described this time as foggy, feeling confused and numb; “I didn’t really feel much at the time. My sickness was pretty bad” [SU 05]. These barriers fundamentally stood in the way of interpreting, processing and sharing their experiences with PODS.

Although patients felt that the discharge form helps to compile information, with a limited view of the process and less reference points than clinicians, it was particularly difficult for them to discuss critiques of the tool, including benefits or barriers to use, or reflect on what could have been better. In discussing their experiences with discharge, they were unable to articulate the problems they encountered specifically with PODS, as it was not distinguished as a separate piece within their entire hospital journey.

## Discussion

To the best of our knowledge, this is the first qualitative study to explore clinicians’ and patients’ experiences of hospital discharge using PODS in a psychiatric hospital setting since its implementation. Although clinicians and patients experience hospital discharges in unique ways, there was substantial agreement between participants in each respective group. Overall, participants felt that PODS helps consolidate important information for clients, their caregivers, and subsequent care clinicians, and serves as a useful reminder for next steps following discharge.

Patients were able to acknowledge the utility of PODS as helpful in organizing certain aspects of their recovery, but not able to evaluate the utility of PODS as a tool. In their discussions, patients focused on their ‘goals of life’, and how their time in the hospital, from admission to discharge, met these goals. This finding supports current literature that patients do not experience services in isolation from each other - their experiences with systems do not start and stop with each service interaction [23, 24].

Although patients faced challenges in providing recommendations to directly improve PODS as a tool, their suggestion to implement “check-in” calls after discharge highlights the need for additional resources to ease transitions and assist with their reintegration into the community. This is particularly important given the patient population, as numerous studies have found that people living with mental illnesses value supported autonomy following discharge [25]. Though helpful, check-in calls may be more challenging to implement in acute care units with high patient turnover and shorter length of stay, and these units will need to be better resourced to provide patients with such options.

Clinicians were able to isolate the benefits and barriers at the system, process and individual level with ease, including contrasting experiences of hospital discharge before and after the implementation of PODS. Clinicians were also able to offer direct recommendations to improve the PODS process, given their role in the implementation of PODS.

It is important to state that the different experiences between patients and clinicians with the PODS process are not necessarily problematic. For clinicians, a hospital discharge is a process that occurs multiple times a week on an inpatient unit. For a patient, a hospital discharge is a unique, personal and sometimes disorienting experience. Although the majority of patients in our study did not experience PODS as a discrete event and were not able to evaluate its efficacy as a tool for discharge planning, that does not detract from the potential benefits of PODS for patients. Unlike a clinician, a patient has less points of comparison for his/her experience of discharge, but nonetheless can benefit from the standardization of the discharge process that occurs as a result of the completion of PODS. Specifically, ensuring information has been transmitted about medication changes, follow-up appointments, and instructions about what to do if one experiences symptoms post-discharge are important facets of discharge planning that may not occur in the absence of the framework PODS provides.

PODS was developed to work in all clinical environments; however, our findings suggest that within a psychiatric hospital setting, PODS may be most suited for units with longer length of stays, as both clinicians and patients have more opportunities to collaborate on their care plan. Perhaps PODS can be introduced by clinicians soon after admission to a unit, to be developed and revised throughout the length of stay.

Since patients’ experiences with services do not start and stop with each interaction, and they view hospital discharge as an integrated part of the service experience, PODS could be implemented at the outset of their journey [25–28]. We propose utilizing journey maps (defined as visualizations of a user experience from start to finish) to illustrate patients’ experiences and interactions with various components of the system as next steps to better understand the challenges and barriers associated with their transition out of the hospital, and highlight key points for intervention.

### Strengths and limitations

We obtained information about clinicians’ and patients’ experiences with hospital discharge using PODS. We only interviewed clinicians and patients at a psychiatric hospital, and their responses are based on their experiences at this hospital and may not reflect the multiple perspectives of individuals at other settings. However, given our rigorous methodological approach to data analysis, we believe that our findings are transferable and can build upon recent study findings from similar patient populations [29–32]. We experienced recruitment challenges with patient participants and found that initiating and maintaining contact with patients who have already discharged from hospital can be difficult. We recommend that future studies begin recruitment well before patients are discharged, as opposed to having clinicians inform them about the study on the day of their discharge, as this time tends to be very busy for both clinicians and patients. It may also be helpful to explore other forms of sampling, such as random sampling, especially within high traffic units, to ensure we are capturing a wide range of patient experiences. Moreover, exploring caregiver perspectives and experiences with PODS would add an additional source of information to triangulate our results given the role of caregivers in mental health care [30, 31]. Finally, to gain a robust understanding of whether PODS continues to meet its goal of helping patients during discharge, it is crucial that subsequent qualitative studies extend the scope to also include patients who did not receive PODS to compare and contrast their experiences.

## Conclusions

This study expands our understanding of hospital discharge from patients’ and clinicians’ perspectives, through evaluating the recently implemented Patient-Oriented Discharge Summaries (PODS) in a psychiatric hospital. The currently available quantitative data indicates that PODS increases patient knowledge of their diagnosis and treatment plan, without overburdening clinicians. However, discussions with clinicians and patients suggest that the discharge process is multifaceted and complex, and though PODS alleviates certain issues, there are many additional areas that need improvement. Moreover, our findings illuminate the barriers of evaluating PODS’ use among patients, and suggest that further work is needed to understand how patients experience discharge. Future research on the impact of PODS on the caregivers’ experience of helping patients transition out of hospital would be an important dimension to study.

## Supplementary information

**Additional file 1.**

## Data Availability

The dataset used and analyzed in the current study are available from the corresponding author on reasonable request.

## Abbreviations

PODS: Patient-Oriented Discharge Summary
CAMH: Centre for Addiction and Mental Health
