# Supplementary material for: Goals of care or goals of life? A qualitative study of clinicians’ and patients’ experiences of hospital discharge using Patient-Oriented Discharge Summaries (PODS)
Source: BMC Health Serv Res. 2020 Jul 24;20:687. doi: 10.1186/s12913-020-05541-7 (PMC7379793; doi:10.1186/s12913-020-05541-7)
Supplement: Supplementary file 1 — Additional file 1. [file 12913_2020_5541_MOESM1_ESM.docx]

**Supplementary Material 1.0 – COREQ Checklist**

| **Topic** | **Item No.** | **Guide Questions/Descriptions** | **Reported on Page No.** |
| --- | --- | --- | --- |
| Domain 1: Research team and reflexivity | | | |
| Personal Characteristics | | | |
| Interviewer/facilitator | 1 | Which author/s conducted the interview or focus group? | 8 |
| Credentials | 2 | What were the researcher’s credentials? E.g. PhD, MD | Credentials provided with author information. |
| Occupation | 3 | What was their occupation at the time of the study? | At the time of the study, all members of the research team were researchers, health professionals/clinicians or scientists. |
| Gender | 4 | Was the researcher male or female? | N/A. |
| Experience and training | 5 | What experience or training did the researcher have? | 9 |
| Relationship with participants | | | |
| Relationship established | 6 | Was a relationship established prior to study commencement? | 7 & 8 |
| Participant knowledge of the interviewer | 7 | What did the participants know about the researcher? E.g. personal goals, reasons for doing the research | 6 |
| Interviewer characteristics | 8 | What characteristics were reported about the interviewer/facilitator? E.g. bias, assumptions, reasons and interests in the research topic | Reflexive statement included in supplementary material 2.0. |
| Domain 2: Study design | | | |
| Theoretical framework | | | |
| Methodological orientation and Theory | 9 | What methodological orientation was stated to underpin the study? E.g. grounded theory, discourse analysis, ethnography, phenomenology, content analysis | 6 |
| Participant selection | | | |
| Sampling | 10 | How were participants selected? E.g. purposive, convenience, consecutive, snowball | 7 |
| Method of approach | 11 | How were participants approached? E.g. face-to-face, telephone, mail, email | 7 & 8 |
| Sample size | 12 | How many participants were in the study? | 9 |
| Non-participation | 13 | How many people refused to participate or dropped out? | N/A |
| Setting | | | |
| Setting of data collection | 14 | Where was the data collected? E.g. home, clinic, workplace | 8 |
| Presence of non-participants | 15 | Was anyone else present besides the participants and researchers? | No. |
| Description of sample | 16 | What are the important characteristics of the sample? E.g. demographic data | Table 1 |
| Data collection | | | |
| Interview guide | 17 | Were questions, prompts, guides provided by the authors? Was it pilot tested? | Interview guide included in supplementary material 3.0. |
| Repeat interviews | 18 | Were repeat interviews carried out? If yes, how many? | No. |
| Audio/visual recording | 19 | Did the research use audio or visual recording to collect the data? | 9 |
| Field notes | 20 | Were field notes made during and/or after the interview or focus group? | No |
| Duration | 21 | What was the duration of the interviews or focus group? | 8 |
| Data saturation | 22 | Was data saturation discussed? | 8 |
| Transcripts returned | 23 | Were transcripts returned to participants for comment and/or correction? | No. |
| Domain 3: Analysis and findings | | | |
| Data analysis | | | |
| Number of data coders | 24 | How many data coders coded the data? | 9 |
| Description of the coding tree | 25 | Did authors provide a description of the coding tree? | Figure 1. |
| Derivation of themes | 26 | Were themes identified in advance or derived from the data? | 9 |
| Software | 27 | What software, if applicable, was used to manage the data? | 9 |
| Participant checking | 28 | Did participants provide feedback on the findings? | No. |
| Reporting | | | |
| Quotations presented | 29 | Were participant quotations presented to illustrate the themes/findings? Was each quotation identified (e.g. participant umber) | Pages 10-20. |
| Data and findings consistent | 30 | Was there consistency between the data presented and the findings? | Pages 21-23. |
| Clarity of major themes | 31 | Were major themes clearly presented in the findings? | Pages 21-23. |
| Clarity of minor themes | 32 | Is there a description of diverse cases or discussion of minor themes? | Pages 21-23. |

Tong A, Sainsbury P, Craig J. Consolidated criteria for reporting qualitative research (COREQ): a 32-item checklist for interviews and focus groups. Int J Qual Health C. 2007; 19(6); 349-57.

**Supplementary Information 2.0 – Reflexive Statement**

Within our research and authorship team, we have a mix of researchers/scientists and health professionals/clinicians. Three of the authors have had substantial involvement with the health care system within various research capacities and/or health service roles. Our collective interactions with the system, as both service providers and service users, have shaped the way we think; we recognize that our experiences have influenced our interpretation of this research and we have had conversations within our team about this, as well as done our own reflective journaling.

**Supplementary Material 3.0 - Interview Guide**

Interview Questions for Service Providers

**Introduction:**

Thank you for agreeing to participate in this interview. We are interviewing you to better understand experiences of hospital discharge. This study will help us understand your experience as a service provider, including your any opinions you have about using a PODS form during discharge and any barriers you might have experienced during the process. There are no right or wrong answers to any of our questions; we are interested in your own experiences.

Participation in this study is voluntary. The interview should take between 25-35 minutes depending on how much information you would like to share. With your permission, I would like to audio record the interview because I don’t want to miss any of your comments. All responses will be kept confidential. This means that your de-identified interview responses will only be shared with research team members and we will ensure that any information we include in our report does not identify you as the respondent. You may decline to answer any question or stop the interview at any time and for any reason.

Are there any questions about what I have just explained?

May I turn on the digital recorder?

**1. Information in the PODS Form**

There are often many instructions and pieces of information that patients must be given during discharge.

- What do you think of the information and instructions that are included in the PODS form?
- What do you like about the form?
- What could be better?

**2. Using the PODS Form**

- What do you think about using the PODS form during discharge?
- What do you like about using the form?
- What could be better?

**3. Barriers to using the PODS Form**

Some patients experience language barriers, illness, or lack of support that can make it hard for them understand or follow discharge instructions.

- What was your experience using the PODS form with patients who might experience these types of barriers?
- Have you ever experienced any personal barriers or difficulties when using a PODS form?

**Prompts:** Please tell me more. Please give me an example.

**Ending Question:** Given what we have discussed, is there anything else that you would like to add? *Thank you very much for your time and the information you shared today.*

Interview Questions for Service Users

**Introduction:**

Thank you for agreeing to participate in this interview. We are interviewing you to better understand what patients think about the discharge instructions they receive in hospital and how they can be improved. There are no right or wrong answers to any of our questions; we are interested in your own experiences.

Participation in this study is voluntary and your decision to participate, or not participate, will not affect the care you receive. The interview should take between 45 minutes to one hour depending on how much information you would like to share. With your permission, I would like to audio record the interview because I don’t want to miss any of your comments. All responses will be kept confidential. This means that your de-identified interview responses will only be shared with research team members and we will ensure that any information we include in our report does not identify you as the respondent. You may decline to answer any question or stop the interview at any time and for any reason.

Are there any questions about what I have just explained?

May I turn on the digital recorder?

**1.  Understanding Discharge Instructions**

For a lot of people, understanding their discharge instructions is a big part of being able to stay out of hospital.

- What did you think of the discharge instructions you were given?
- What did you like best?
- What could be better?

**Prompts**: Please tell me more.  Please give me an example.

**2. Use of Discharge Instructions**

Some people see discharge instructions as a way to achieve their goals for living at home.

- How did you use your discharge instructions when you got home?

**Prompts:**Please tell me more.  Please give me an example (facilitator guides to each section of discharge instructions and asks for examples).

**3. Factors affecting the ability to use the instructions**

Some people experience language barriers, illness, or lack of support as things that can make it hard for them to use discharge instructions.

- What was your experience with these types of barriers?

**Prompts:** Please tell me more.  Please give me an example.

**4. Use of Discharge Instructions by Family or Other Support Systems**

Some people have family members or other people assist them in following their discharge instructions.

- If you received assistance in this way, did this person or these people share any opinions about your discharge instructions?
- What did they like best?
- What could be better?
- Did they experience any barriers in helping you follow your discharge instructions?

**Prompts:**Please tell me more. Please give me an example.

**Ending Question:** Given what we have discussed, is there anything else that you would like to add? *Thank you very much for your time and the information you shared today.*
